# Essential Roles of GABA Transporter-1 in Controlling Rapid Eye Movement Sleep and in Increased Slow Wave Activity after Sleep Deprivation

**DOI:** 10.1371/journal.pone.0075823

**Published:** 2013-10-14

**Authors:** Xin-Hong Xu, Wei-Min Qu, Min-Juan Bian, Fang Huang, Jian Fei, Yoshihiro Urade, Zhi-Li Huang

**Affiliations:** 1 Department of Pharmacology, Shanghai Medical College, Fudan University, Shanghai, China; 2 Institutes of Brain Science, Fudan University, Shanghai, China; 3 State Key Laboratory of Medical Neurobiology, Fudan University, Shanghai, China; 4 School of Life Science and Technology, Tongji Universities, Shanghai, China; 5 Department of Molecular Behavioral Biology, Osaka Bioscience Institute, Suita, Japan; Kent State University, United States of America

## Abstract

GABA is the major inhibitory neurotransmitter in the mammalian central nervous system that has been strongly implicated in the regulation of sleep. GABA transporter subtype 1 (GAT1) constructs high affinity reuptake sites for GABA and regulates GABA*ergic* transmission in the brain. However, the role of GAT1 in sleep-wake regulation remains elusive. In the current study, we characterized the spontaneous sleep-wake cycle and responses to sleep deprivation in GAT1 knock-out (KO) mice. GAT1 KO mice exhibited dominant theta-activity and a remarkable reduction of EEG power in low frequencies across all vigilance stages. Under baseline conditions, spontaneous rapid eye movement (REM) sleep of KO mice was elevated both during the light and dark periods, and non-REM (NREM) sleep was reduced during the light period only. KO mice also showed more state transitions from NREM to REM sleep and from REM sleep to wakefulness, as well as more number of REM and NREM sleep bouts than WT mice. During the dark period, KO mice exhibited more REM sleep bouts only. Six hours of sleep deprivation induced rebound increases in NREM and REM sleep in both genotypes. However, slow wave activity, the intensity component of NREM sleep was briefly elevated in WT mice but remained completely unchanged in KO mice, compared with their respective baselines. These results indicate that GAT1 plays a critical role in the regulation of REM sleep and homeostasis of NREM sleep.

## Introduction

Brain function is based on an exquisite balance between excitatory and inhibitory neurotransmission. Gamma-aminobutyric acid (GABA) is the principal inhibitory neurotransmitter in the mammalian nervous system, where it actives GABA_A_, GABA_B_ and GABA_C_ receptors. GABAergic inhibitory mechanisms are crucial for the initiation and maintenance of sleep [1]. GABA*ergic* processes are responsible for rapid eye movement (REM) sleep occurrence. Injection of the GABA_A_ receptor (GABA_A_R) antagonists bicuculline or GABAzine into the sublaterodorsal nucleus (SLD), REM-on neurons induces a REM-like state in rats and cats [2], [3], [4]. In contrast, inactivation of the ventrolateral part of the periaqueductal gray (VLPAG) and the adjacent dorsal part of the deep mesencephalic reticular nuclei (dDpMe) REM-off neurons by muscimol (a GABA_A_ agonist) application induces strong increases in REM sleep quantities [5], [6]. The regions responsible for the generation of non-REM (NREM) sleep are located in the ventrolateral preoptic area (VLPO) and/or median preoptic nucleus (MnPO), where neurons showing sleep related c-Fos immunoreactivity are identified GABA*ergic* [7], [8]. Nitz and Siegel [9] reported that extracellular level of GABA in the posterior hypothalamus is increased in the NREM sleep and is lowered while awake. In addition, it is well established that activation of GABA_A_R favors sleep. Three generations of hypnotics are based on these GABA_A_R-mediated inhibitory processes.

Although the important role for GABA in the sleep–wake cycle is commonly accepted, many studies in this field were focused on the role of GABA receptors; less is known about the function of GABA transporters (GATs) in regulation of the sleep-wake. Plasma membrane GATs contribute to determining GABA levels in the synaptic cleft and extracellular space [10], [11]. Through the reuptake of released GABA, GATs control the duration and intensity of GABAergic activity at the synapse. Molecular cloning studies have identified multiple GAT subtypes, including GAT1, GAT2, GAT3, GAT4, and vesicle GAT. Among them, GAT1 is the major subtype distributed at both synaptic and extrasynaptic sites in the brain, including the hippocampus, neocortex, cerebellum, and retina [11], [12]. Uptake assay showed that >75% of GABA uptake is contributed by GAT1 in the central nervous system [13].

Several significant alterations of GABAergic transmission have been investigated in electrophysiological experiments using GAT1 knockout (KO) mice. In neocortex and hippocampus, GAT1 deficiency leads to a large increase in a tonic postsynaptic GABA_A_R-mediated conductance, but little or no change in the amplitude and shape of spontaneous IPSCs [13], [14]. Chronically elevated GABA levels also down-regulate phasic GABA release and reduce presynaptic signaling via GABA_B_R [13]. Behavioral tests showed that GAT1 deficiency causes impaired rotarod performance, and reduced locomotor activity in the home cage [15]. GAT1 KO mice showed a lower level of anxiety-like behaviors and decreased insensitivity to both the sedative/hypnotic drugs [16]. These findings indicated GAT1 may be involved sedative/hypnotic effect. Tiagabine, a selective GAT-1 inhibitor has been used clinically as add-on treatment for epilepsy [17]. Somnolence has been reported as a side effect of tiagabine [18], and was tested for its sedative potential in rats and humans [19], [20]. Indeed, tiagabine has been shown to improve the efficiency of sleep in rats and to enhance sleep in healthy elderly people. However, tiagabine also has also been found to have affinity for the histamine H_1_ receptor and the benzodiazepine sites [21], which may contribute to its sedative–hypnotic effects. In addition, GAT1 inhibitors as a pharmacological tool to determine GAT1 functions exhibit limited selectivity and/or incomplete blockade. In comparison, GAT1 KO mice can provide more accurate assessment of the contribution of this transporter to sleep–wake regulation.

We hypothesized that the congenital lack of GAT1, potentially involved in the regulation of sleep, could lead to altered sleep patterns. We report that GAT1 KO mice spent longer time in REM sleep and shorter time in NREM sleep during the light period. After 6-h sleep deprivation, the slow wave activity of NREM sleep increased transiently in WT mice but remained unchanged in the KO mice. These results indicate that GAT1 plays pivotal roles in the regulation of REM sleep and homeostasis of NREM sleep.

## Materials and Methods

### Animals

Male GAT1 KO mice and their WT littermate controls of the inbred C57BL/6 strains from heterozygotes were generated as previously described [22] and used for the current experiments at the State Key Laboratory of Medical Neurobiology, Fudan University (Shanghai, China). The animals were housed in an insulated and soundproof recording room that was maintained at an ambient temperature of 22±0.5°C with a relative humidity of 60±2% on an automatically controlled 12-h light/dark cycle (lights on at 07:00, illumination intensity≈100 lux). Food and water were given ad libitum. Experimental protocols were approved by the Medical Experimental Animal Administrative Committee of Shanghai, in accordance with Guide for the Care and Use of Laboratory Animals (The National Academies Press, 8th Ed. Washington D.C., 2011). Every effort was made to minimize the number of animals used and any pain or discomfort experienced by the animals.

### Polygraphic recordings

Under chloral hydrate anesthesia (360 mg/kg, i.p.), mice were chronically implanted with electroencephalogram (EEG) and electromyogram (EMG) electrodes for polysomnographic recordings. The implant consisted of two stainless steel screws (1 mm in diameter) that were inserted through the skull (anteroposterior, +1.0 mm; medio-lateral −1.5 mm from bregma) according to the mice brain atlas [23] and served as EEG electrodes. Two Teflon-coated, insulated stainless steel wires were bilaterally placed into both trapezius muscles and served as EMG electrodes. All the electrodes were attached to a microconnector and fixed to the skull by dental cement. The EEG and EMG recordings were carried out by a specifically designed slip ring that would not restrict the movement of the mice.

After a 7-day recovery period, the mice were housed individually in transparent barrels and habituated to the recording cable for 3–4 days before polygraphic recording. For the study of spontaneous sleep–wakefulness cycles, each animal was recorded for 24 h continuously beginning at the onset of the light period (7:00 A.M.).

Cortical EEG and EMG signals were amplified and filtered (EEG, 0.5–30 Hz; EMG, 20–200 Hz) and then digitized at a sampling rate of 128 Hz and recorded by SleepSign (Kissei Comtec, Nagano, Japan) as previously described [24], [25], [26], [27].

### Vigilance state judgment and EEG spectral analysis

The sleep-wake states were automatically classified by 10-s epochs as wake, NREM, or REM sleep by SleepSign 2.0 according to published standard criteria [24], [28], [29], [30], [31]. The defined sleep-wake stages were then examined visually and corrected if necessary. Each stage was characterized as follows: NREM sleep, high-amplitude slow or spindle EEG, and low-voltage EMG activities; REM sleep, low-voltage EEG and EMG activities. Spectral characteristics of the EEG were further analyzed offline. We used fast Fourier transform (FFT) to obtain absolute EEG power spectra for each animal and each behavioral state.

To obtain a reference value, total state-specific power was computed from the averaged spectra by summarizing all frequency bins from 0 to 24.5 Hz. The power of each 0.5 Hz bin was first averaged across the sleep stages individually and then normalized as a group by calculating the percentage of each bin from the total power (0–24.5 Hz) of the individual animal.

### Sleep deprivation

To examine the integrity of sleep homeostasis, all mice were subjected to SD. SD was performed by gently tapping on the cage via a soft tissue ball whenever the animals looked drowsy or the EEG showed signs of low-frequency activity. Mice were recorded for two consecutive days for EEG and EMG. The first day served as the baseline. On the second day served as experimental day, the animals were subjected to total SD for 6 h (from 13:00 to 19:00). A subsequent undisturbed 18-h period was recorded to assess the dynamics of sleep recovery in mice.

To measure the effects of sleep deprivation (SD) on EEG power, the slow wave activity (SWA, 0.5–4 Hz) and theta activity (TA, 6–10 Hz) in NREM or REM sleep were compared between the night after SD ended and the corresponding baseline night.

### Statistical analysis

All results were expressed as means ± SEM. For vigilance studies, unpaired *t-*test was used for statistical comparison between two genotype groups. Differences between the baseline and experimental day or genotype differences in SD treatment were compared using a two-way ANOVA (with genotype and SD treatment as factors). *Post hoc* paired or unpaired *t* test were performed if the results of the ANOVA reached statistical significance (p<0.05).

## Results

### GAT1 KO mice showed exaggerated EEG theta-activity and a remarkable reduction of EEG power in low frequencies

**Figure 1:**
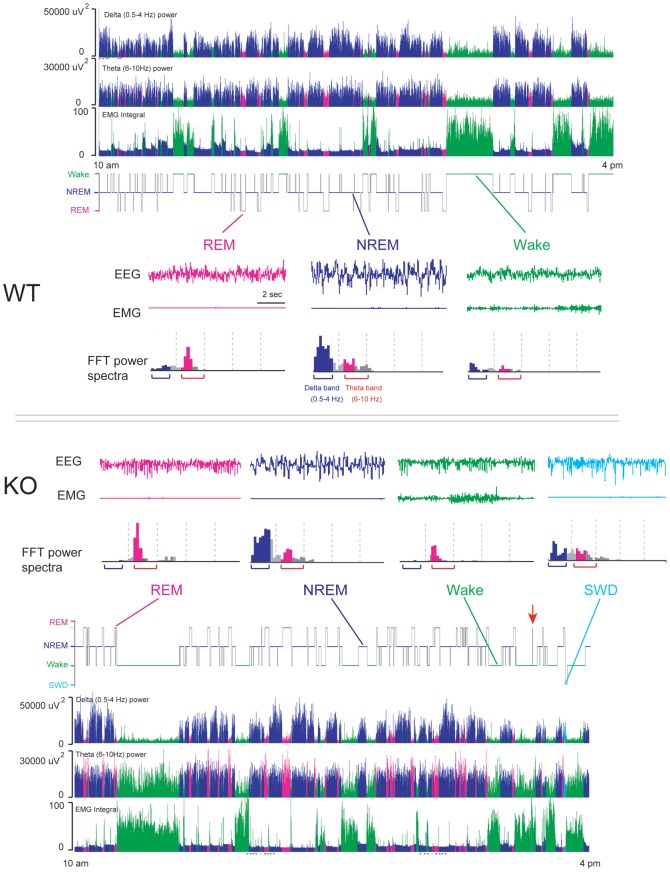
GAT1 KO mice displayed abnormal EEG activity. Representative examples for hypnogram, FFT-derived delta (0.5–4 Hz) power, FFT-derived theta (6–10 Hz) power and EMG activity in a WT (above) and KO mouse (below) from 10 am to 4 pm, as well as typical examples of EEG/EMG polygraphic recordings in each vigilance stage and corresponding power spectrum for 10-sec epochs for both genotypes. SWD, spike-wave discharge.

Figure 1 showed representative examples hypnogram, FFT-derived delta (0.5–4 Hz) power, FFT-derived theta (6–10 Hz) power and EMG activity over 6 h starting from 10:00 to 16:00 during the light period for a WT and a GAT1 KO mouse, respectively. During NREM sleep, FFT-derived delta (0.5–4 Hz) power was greater than FFT-derived theta (6–10 Hz) power. However, low integrated values of the delta frequency band and high integrated values of the theta frequency band were observed during REM sleep. During NREM-REM transitions, GAT1 KO exhibited a sharp decrease in the FFT-derived delta power, meanwhile, increase of the FFT-derived theta power can be observed during REM sleep.

Figures 1 also showed representative examples of 10-sec raw EEG and EMG traces in each vigilance stage for both genotype. In WT mice, their wake epoch was characterized by desynchronized low-amplitude EEG accompanied by a sustained EMG activity. Their NREM sleep epoch was clearly distinguished by high voltage slow waves and spindles. Integrated values for the delta frequency band were greater than those for the theta frequency band. They also showed low-voltage EMG activities. Their REM sleep epoch showed low amplitude EEG, low integrated values of the delta frequency band, and lower-voltage EMG activities (Figures 1, above). The EEG of KO mice, by contrast, was dominated by theta-activity and much higher integrated values of the theta frequency band in both REM and NREM sleep or even when they were awake (Figures 1, below). A previous study showed that these KO mice display readily observable, nearly continuous tremor in the limbs and tail, and the tremor frequency is 25–32 Hz [15]. Therefore, EMG activities were very high during wakefulness so that there were no obvious quiet wake episodes. And, integrated values of theta power of WT mice were low, but many those in GAT1 KO mice were high during wakefulness. These results indicated that GAT1 deficiency exaggerated EEG theta-activity.

Another unexpected finding was the occurrence of spike-wave discharge (SWD) in all KO mice. Their SWD epoch showed some abrupt EEG sharp wave, high integrated values of the delta and theta frequency band, and lower-voltage EMG activities. Over the 24 h baseline recording period, mean numbers of SWD in KO mice was 2.7 and lasted about 27.5 sec.

**Figure 2:**
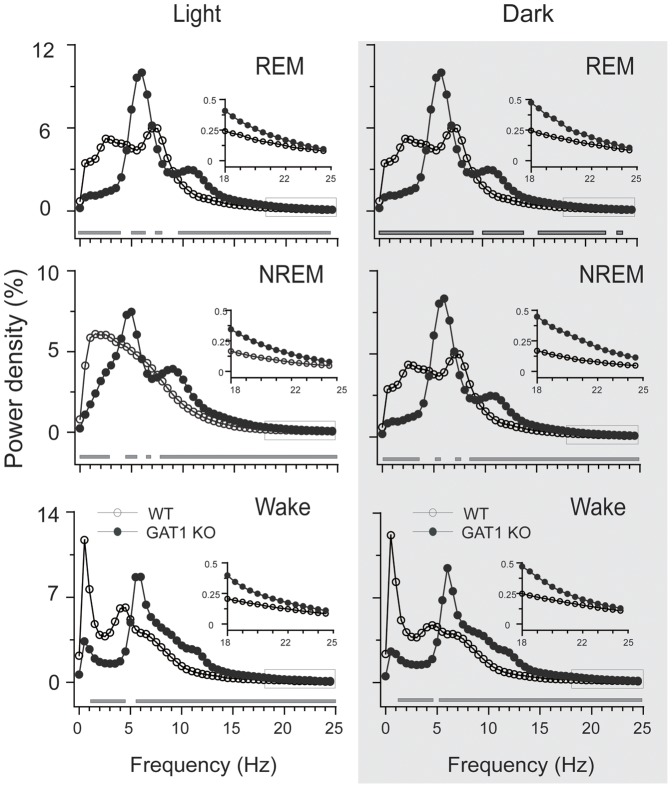
GAT1 KO mice displayed dominant EEG theta-activity. EEG power density curves for each stage during light (left panel) and dark (right panel) periods. The horizontal bars indicate where there is a statistical difference (P<0.01) between the WT (n = 7) and KO mice (n = 8), assessed by unpaired *t*- test.

Spectral analysis of EEG power density in each vigilance state revealed that GAT1 KO mice displayed a remarkable reduction of EEG power in low frequencies (<4 Hz) and much higher theta (4–10 Hz) activity, and a significant increase in the high frequency range compared with the WT mice (Figure 2). During REM sleep, the peak of the EEG power density curve (theta oscillation) was left-shifted from 7.5 Hz in the WT mice to 6.0 Hz in the KO mice, as previously reported by Gong et al. [32]. During NREM sleep, the peak of the EEG power density curve (0.5–4 Hz in WT mice) was masked in the KO mice by the increase and shift of the theta frequency band. These results indicated that GAT1 was involved in theta and delta activities.

### GAT1 KO mice showed greater amounts of REM sleep

**Figure 3:**
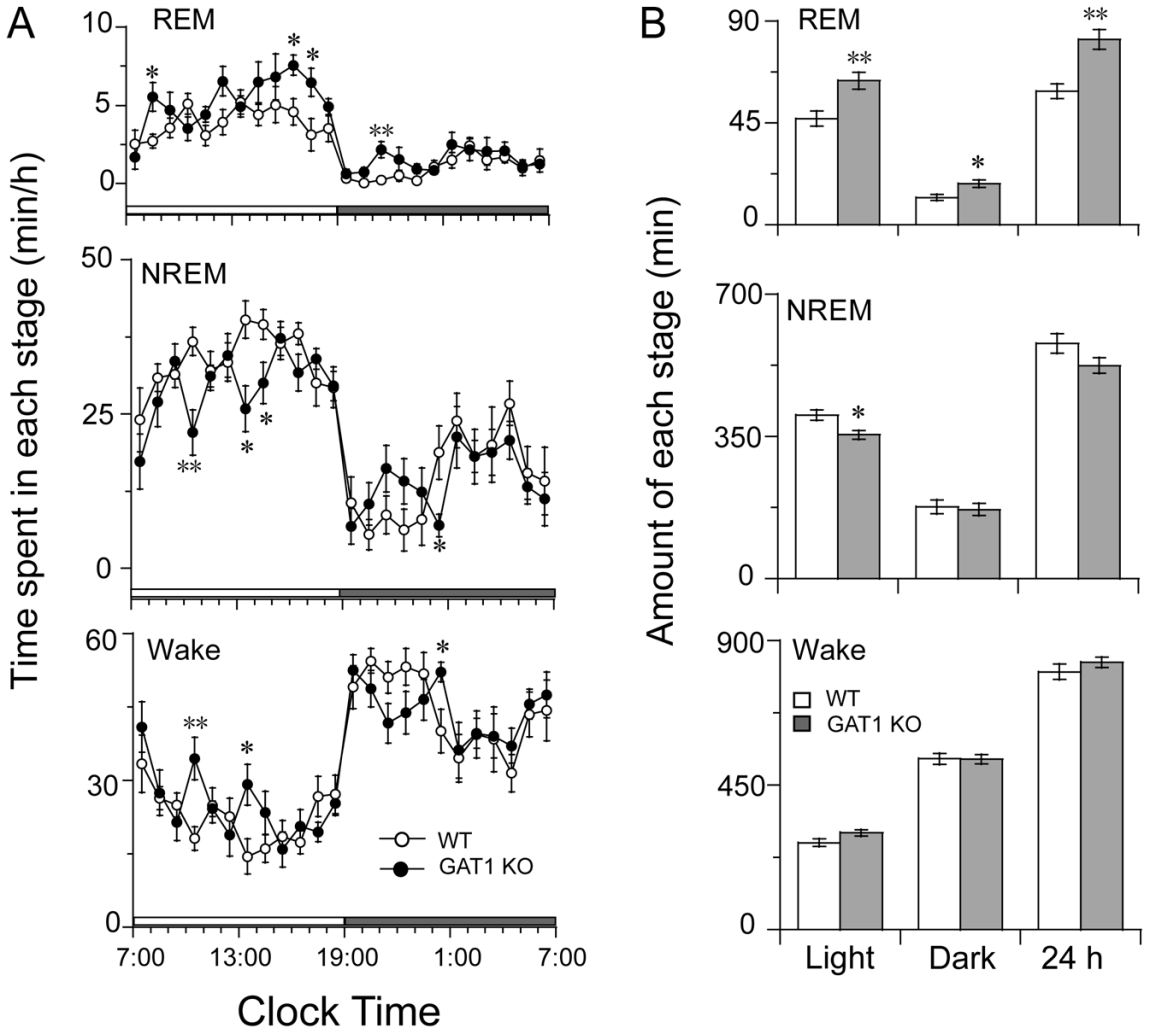
Sleep-wake profiles of the WT and GAT1 KO mice under baseline conditions. (A) Time-course changes in each stage. Each circle represents the hourly mean amount of each stage. Open and filled circles stand for the values of the WT and KO mice, respectively. The horizontal open and filled bars on the χ-axis indicate the 12-h light and 12-h dark periods. (B) Total time spent in each stage for 12-h light/dark periods and 24 h all day. Open and filled columns represent the profiles for the WT (n = 7) and KO (n = 8) mice, respectively. Values are expressed as means ± SEM. * P<0.05 and ** P<0.01, compared with the corresponding WT control, assessed by unpaired *t*-test.

Under baseline conditions, both GAT1 KO (n = 8) and WT (n = 7) mice exhibited a clear circadian sleep–wake rhythm with increased amounts of sleep during the light period than the dark period. Compared with the WT mice, GAT1 KO mice showed greater amounts of REM sleep and a reduced NREM sleep during the light period (Figure 3). Unexpectedly, we found that a few REM sleep epochs occurred between wakefulness epochs in KO mice (showed as “red arrow” in Figure 1). It meant that REM sleep starts directly from wakefulness. When total amounts of REM and NREM sleep were calculated during 12-h light or dark periods, GAT1 KO mice exhibited significantly increased REM sleep by 35.7% and 50.5% [light: *t* (13) = 3.350, p = 0.005; dark: *t* (13) = 2.875, p = 0.013] and decreased NREM sleep by 12.1% [light: *t* (13) = 2.968, p = 0.011] compared with WT mice (Figure 3B). However, there was no difference in wakefulness between the two genotypes.

### GAT1 KO mice showed more numbers of REM sleep bouts than the WT

#### Mice

**Figure 4:**
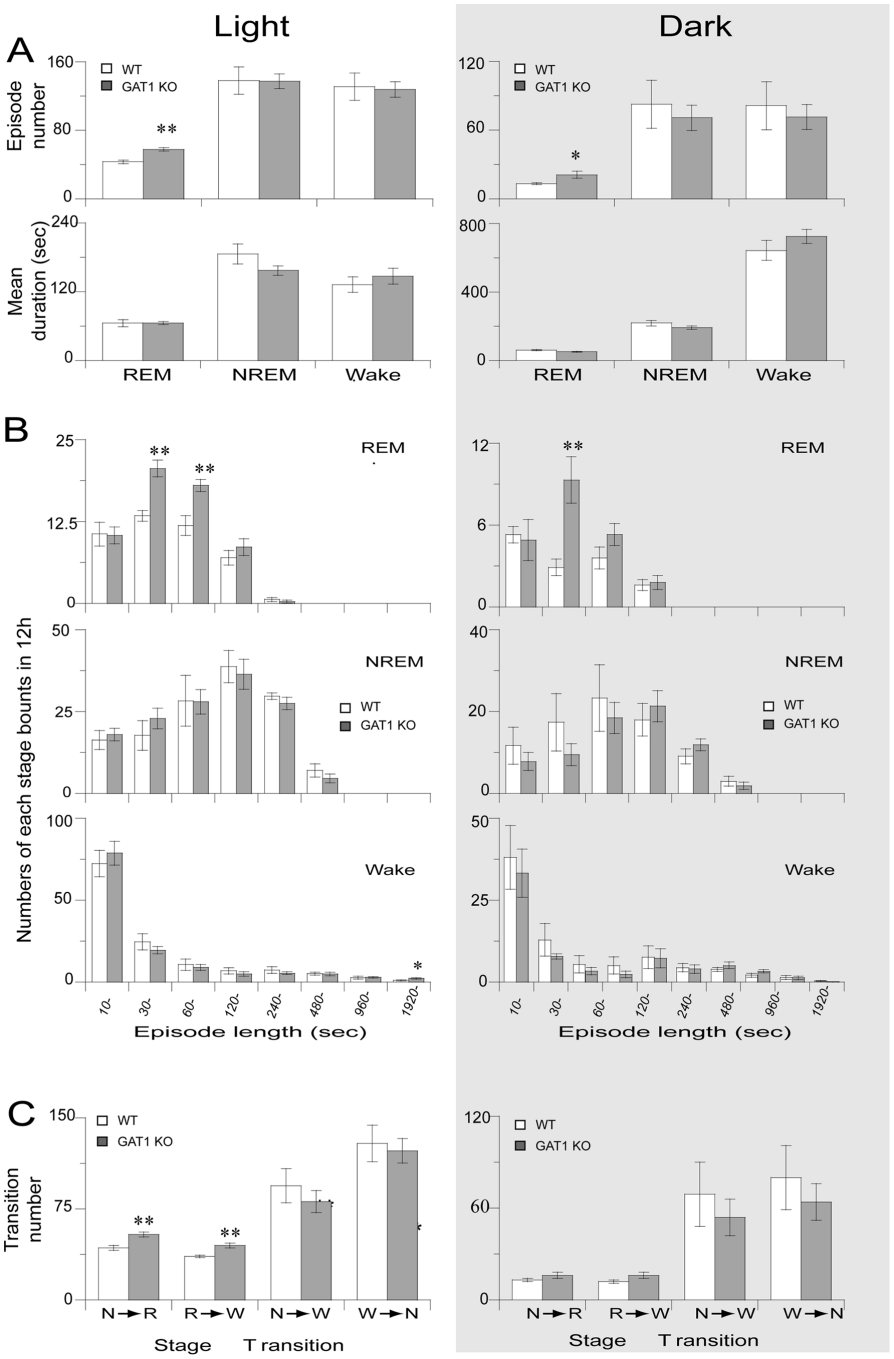
GAT1 KO mice showed more sleep bouts and stage transition than the WT mice. (A) Episode numbers and mean durations. (B) Numbers of sleep bouts in 12 h. (C) Stage transitions during 12-h light and 12-h dark phases. Open and filled columns represent the profiles for the WT and GAT1 KO mice, respectively. Values are expressed as means ± SEM. * P<0.05 and ** P<0.01, compared with the corresponding WT control, assessed by unpaired *t*-test.

The episode numbers and mean duration of three vigilance stages are summarized in Figure 4A. Compared with WT mice, REM sleep episode number of GAT1 KO mice increased by 1.3-fold in GAT1 KO mice [*t* (13) = 5.016, p<0.001] during the light period.. During the dark period, REM bouts increases by 1.6-fold in KO mice [*t* (13) = 2.479, p = 0.039]. There was no difference was observed in the mean duration between these two genotypes. GAT1 KO mice showed unchanged episode numbers and mean durations of NREM and wakefulness relative to WT mice. We further calculated the distribution of REM sleep and found that KO mice particularly had more REM sleep bouts in the ranges of 30–60 and 60–120 sec during the light period and in the ranges of 30–60 sec during the dark period than WT mice (Fig. 4B). These results suggested that enhanced GABA transmission can help the maintenance and induction of REM sleep. The distribution of NREM bouts and wakefulness did not show significant changes between these two genotypes, except increased NREM bouts in the ranges of >1920 sec during the light period.

Stage transition analysis showed that the numbers of transitions in the light period from NREM to REM and from REM to wakefulness increased by 20% [t (13) = 4.118, p = 0.001] and 19.3% [*t* (13) = 3.360, p = 0.005], respectively. However, no difference was observed during the dark period between these two genotypes.

### GAT1 KO mice showed no transient increase in SWA after sleep deprivation

**Figure 5:**
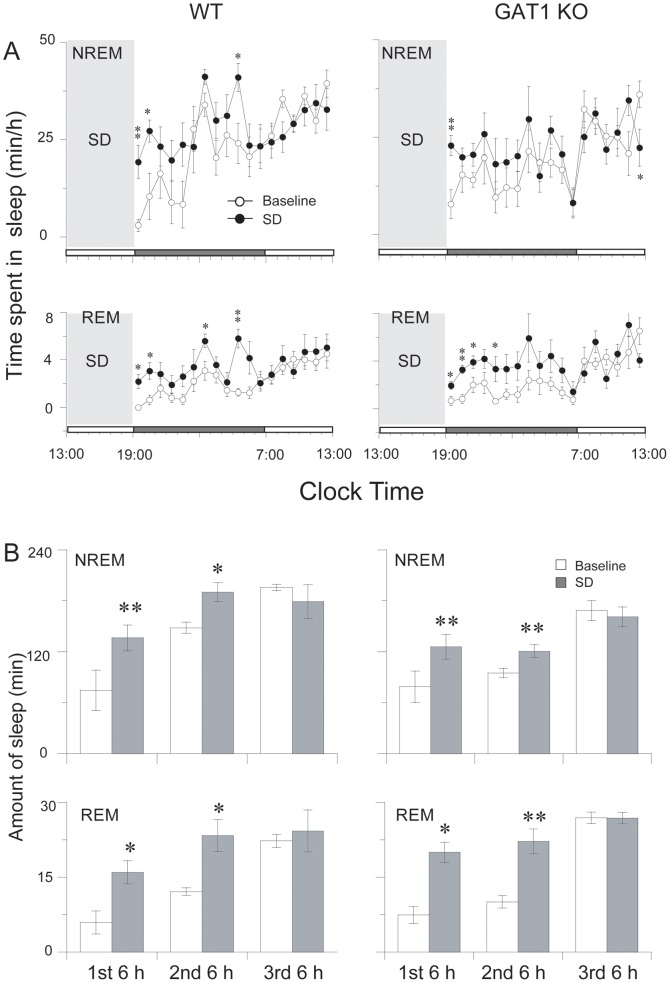
Effects of sleep deprivation (SD) on sleep-wake profiles. (A) Time course changes of NREM and REM sleep in the WT (n = 6) and GAT1 KO (n = 7) mice. Each circle represents the hourly mean amount of sleep. Open and filled circles stand for the baseline and SD profiles, respectively. The horizontal open and filled bars on the χ-axis indicate the 12-h light and 12-h dark periods. (B) Total amount of NREM and REM sleep for 12 h in darkness after 6 h SD compared with corresponding baseline. Values are expressed as means ± SEM. Differences between the baseline and experimental day:* P<0.05 and ** P<0.01, paired *t*-test was performed if the results of the two-way ANOVA reached statistical significance.

To determine the role of GAT1 in the homeostatic regulation of sleep, we applied 6-h SD from 13:00 to 19:00 to compare the rebound sleep between WT (n = 6) and GAT1 KO mice (n = 7). As shown in Figure 5A, a two-way ANOVA revealed that SD treatment had significantly effects on NREM sleep [1^st^ 6 h: F (3, 20) = 8.726, p = 0.008; 2^nd^ 6 h: F(3,20) = 17.938, p<0.001] and REM sleep [1^st^ 6 h: F (3, 20) = 29.256, p<0.001; 2^nd^ 6 h: F(3, 20) = 29.175, p<0.001] in two genotype mice. For response to SD (Figure 5B), there was no genotype difference [1st 6 h NREM sleep: F (3, 20) = 0.031, p = 0.863; 1st 6 h REM sleep: F (3, 20) = 1.731, p = 0.203; 2nd 6 h REM sleep: F (3, 20) = 0.053, p = 0.175], except the second 6 h NREM sleep [F (3, 20) = 59.8, p<0.001]. High baseline level in some WT mice resulted in genotype difference in 2nd 6 h NREM sleep. In the first 6 h interval, the total amounts of NREM and REM sleep increased from the baseline by 1.8-fold [*t* (5) = 4.775, p = 0.005] and 2.7-fold [*t* (5) = 3.574, p = 0.016]in the WT mice and by 1.6-fold [*t* (5) = 6.548, p = 0.001] and 2.7-fold [*t* (5) = 3.332, p = 0.021], respectively. In the second 6 h interval, the total amounts of NREM and REM sleep increased from the baseline by 1.3-fold [*t* (5) = 3.574, p = 0.016] and 1.9-fold [*t* (5) = 3.465, p = 0.018] in the WT mice and by 1.3-fold [*t* (5) = 8.294, p<0.001] and 2.2-fold [*t* (5) = 6.394, p = 0.001] in the KO mice, respectively.

**Figure 6:**
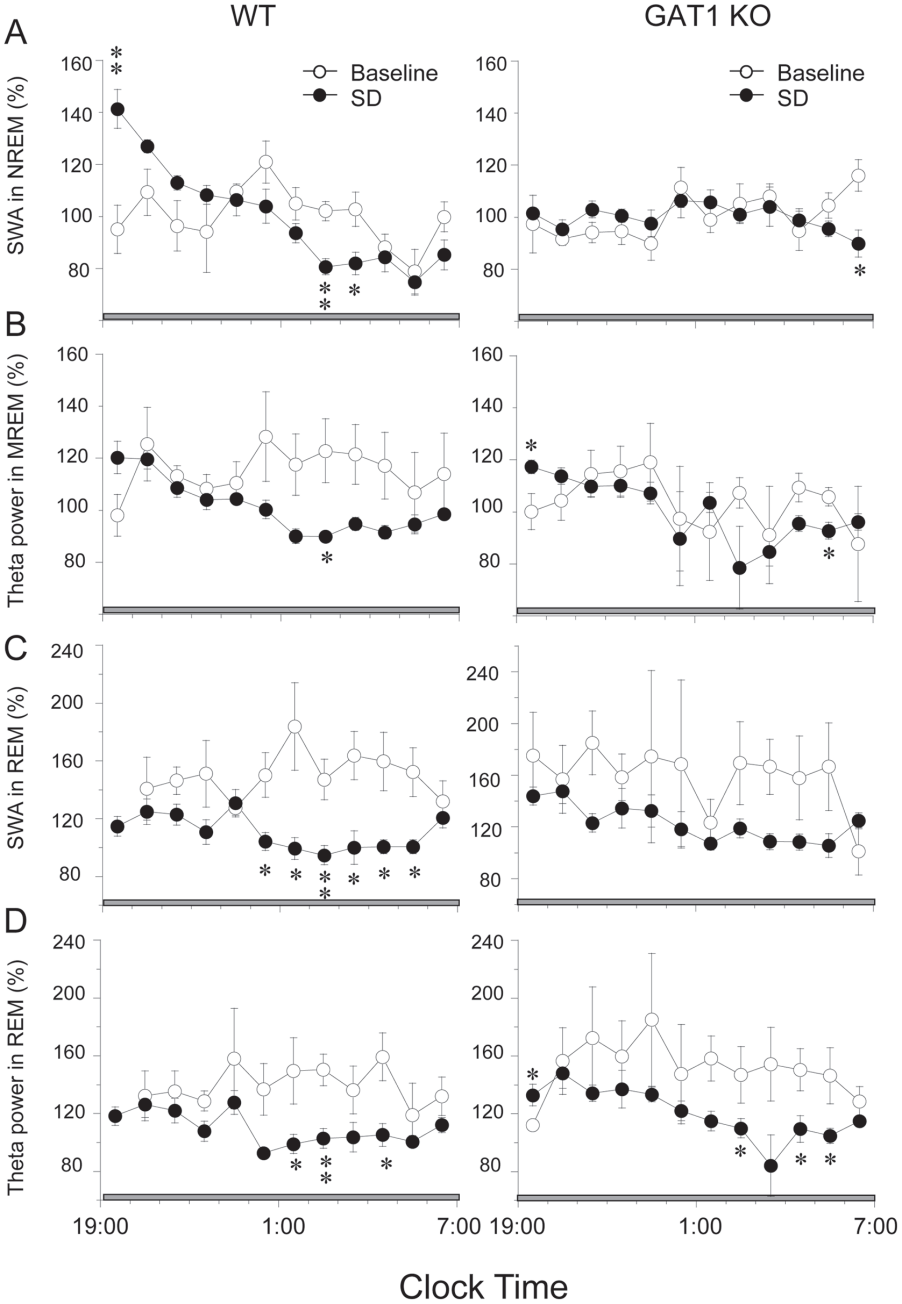
Effects of sleep deprivation (SD) on EEG power. Time course of slow wave activity (SWA) and theta activity in NREM or NREM in WT and GAT1 KO mice during 12-h recovery after 6-h SD. Mean hourly values are expressed as percentage of mean 24-h baseline SWA or theta activity in NREM or REM sleep of the corresponding EEG. Values are expressed as means ± SEM. * P<0.05 and ** P<0.01, compared with the corresponding baseline, assessed by paired *t*-test.

SWA within NREM sleep encompassing the frequency band between 0. 5 and 4 Hz was observed after prolonged periods of wakefulness. Immediately after SD, the SWA in NREM sleep increased for 2 h and then gradually returned to the baseline level in the WT mice, whereas this transient increase in SWA was abolished in the KO mice (Figure 6A). Meanwhile, we examined the SWA in REM sleep and found that the SWA in REM sleep decreased from 0:00 to 5:00 in WT mice, whereas this transient decrease was not significant in the KO mice (Figure 6C). Response to SD, the theta activity in NREM or REM sleep was similar in two genotype groups (Figure 6B, D). The absence of rebound SWA is abnormal and reflects an altered homeostasis in the KO mice, indicating that GAT1 plays a pivotal role in the homeostatic regulation of NREM sleep.

## Discussion

Electrical activity of the brain is determined by specific interactions between billions of excitatory and inhibitory neurons of the neocortex and subcortical structures. All major oscillations in the electroencephalogram (EEG), namely delta, theta, spindle and gamma frequencies, arise from synchronous neuronal activity in the neocortex, thalamus, hippocampus and other brain structures, where GABAergic interneurons may play an important role [33], [34].

According to spectral analysis of EEG power density, we found that GAT1 deficiency increased theta-activity in the stage of REM and NREM sleep, even wakefulness. Theta oscillations characterized with high voltage and low frequency (in rodents 6–9 Hz). The medial septum and diagonal band of Broca (MS-DBB) has been classically viewed as the hippocampal theta rhythm generator [35], where cholinergic and GABAergic neurons are well known to exist. In vivo, cholinergic neurons discharge infrequently while GABAergic neurons produce rhythmic bursts phase locked to the ongoing hippocampal theta oscillations [36], [37]. GAT 1 is the most abundantly expressed GAT in the brain and is particularly plentiful in the area rich in GABA*ergic* neurons, such as the hippocampus, neocortex, cerebellum, and retina [11], [12]. Deficiency of GABA reuptake inevitably decreases the rate of GABA clearance from synaptic cleft and enhances the basal extracellular concentration of GABA. Jensen et al. [13] observed a large increase in a tonic postsynaptic hippocampal GABA_A_ receptor-mediated conductance in GAT1 KO mice. Therefore, an elevated GABA level may persistently activate GABA receptors, and increase hippocampal theta activity in GAT1 KO mice.

On the other hand, the frequency at which theta power reached maximum in the GAT1 KO mice was significantly left-shifted from 7.5 Hz to 6.0 Hz, although this peak frequency remained in theta band. This result is consistent with the previously reported by Gong et al. [32]. Thus, GAT1 activity does not affect the physiological expression of theta oscillation activity, but modulates the precise frequency of this oscillation. Another unexpected finding was the occurrence of spike-wave discharge (SWD) in KO mice. This deviant morphology looks like the EEG profiles of spontaneous seizures in GABA_B_ receptors KO mice [38]. These mice devoid of functional GABA_B_ receptors also exhibited the increase in theta power during wakefulness. An earlier study showed that GABA_B_ antagonists consistently decreases low-frequency EEG activity during NREMS in cats [39]. In the present study, we also found a remarkable reduction of EEG power in low frequencies (<4 Hz). These findings indicated that the function of GABA_B_ receptors were down-regulated in GAT1 KO mice. An electrophysiological study by using GAT1 KO mice reported that chronically elevated GABA levels can down-regulate phasic GABA release and reduce presynaptic signaling via GABA_B_R in the hippocampus [13], suggesting that the EEG of the KO mice reflects an abnormal activity that also may be mediated by down-regulation of the function of GABA_B_ receptors in GAT1 KO mice.

Although no consensus has yet emerged regarding their specific behavioral correlates, theta waves are most consistently present during REM sleep [40]. In the present study, we found that the GAT1 KO mice spent longer time in REM sleep with more state transitions from NREM to REM sleep and longer REM bouts, suggesting that enhanced GABA transmission can help the maintenance and induction of REM sleep. However, the brain regions involved in the action of GAT1 still remain unknown. It has then been shown that, a mutual inhibitory interaction between GABAergic sublaterodorsal nucleus (SLD) REM-on and GABAergic the ventrolateral part of the periaqueductal gray (VLPAG) and the adjacent dorsal part of the deep mesencephalic reticular nuclei (dDpMe) REM-off neurons constitutes a brainstem flip-flop switch controlling REM sleep onset and maintenance [41]. However, the presence of c-FOS/GAD67 double-labeled neurons cannot be confirmed in the SLD in REM sleep hypersomniac animals. Recently study showed that the lateral hypothalamic area (LH) is the only brain structure containing a very large number of neurons activated during REM hypersomnia and projecting to the VLPAG/dDpMe. After muscimol injections in the LH, the VLPAG/dDpMe contained a large number of activated neurons, mostly GABAergic, and projecting to the SLD [5]. These results strongly suggest that these neurons trigger REM sleep by means of their inhibitory projection to the PS-off GABAergic neurons located in the VLPAG/dDpMe. Although we speculate that the observed REM sleep phenotype is due to increased GABA signaling in the brainstem REM sleep-regulatory pathways, this question may ultimately be answered by the focal knockout of GAT1 in the VLPAG/dDpMe. In addition, we found that the onset of REM sleep was directly from wakefulness in KO mice. This finding indicated orexinergic system may have been modified in GAT1 mutant mice.

Studies suggest that the intensity of SWA in the cortical EEG is the single most important process for the homeostatic regulation of NREM sleep [42], [43], [44]. In the present study, no difference in the amount of sleep rebound was observed between the 2 genotypes, whereas a transient increase of SWA in the NREM sleep that occurred in WT mice was abolished in the KO mice. These alterations indicated that the absence of rebound SWA is abnormal and reflects an altered homeostasis in GAT1 KO mice, suggesting that GAT1 plays a pivotal role in the homeostatic regulation of NREM sleep.

Gvilia, et al. provide the first evidence that the activity of the MnPO and VLPO neurons is related to the need for sleep and that these neurons may constitute part of the forebrain circuitry involved in the homeostatic regulation of sleep [45]. These results indicate that MnPO GABA*ergic* neurons are strongly activated in response to increasing sleep pressure, whereas VLPO GABA*ergic* neurons are activated in response to increasing sleep amount. We presumed that a neuroadaptation process could occur in the GABA*ergic* system after its chronic exposure to a high level of GABA level. The GAT1 deficiency leads to enhanced postsynaptic tonic conductance in the VLPO, resulting in sleep rebound after SD, whereas chronically elevated GABA levels could down-regulate phasic GABA release and attenuate activation of GABA_B_ receptors in MnPO, resulting in SWA suppression.

In summary, GAT1 KO mice showed increased theta-activity in each vigilance stage, spent longer time in REM sleep, and experienced more state transitions from NREM to REM sleep and longer REM bouts during the light period than the WT mice. These results indicate that GAT1 plays pivotal roles in the regulation of REM sleep and homeostasis of NREM sleep. In addition, GAT1 KO mice may be a valuable tool for the elucidation of GABAergic system on the regulation of cortical synchronization of neuronal activity and suggest a link between regional EEG synchronization and behavioral states. The problem with the results obtained is that it is not possible to determine which types of GABAergic neurons are responsible for the phenotype observed. It would require local and temporary inactivation of GAT1.
