# Correction: Essential Roles of GABA Transporter-1 in Controlling Rapid Eye Movement Sleep and in Increased Slow Wave Activity after Sleep Deprivation

**DOI:** 10.1371/annotation/043edac7-b29e-40d8-b7aa-017d6a3a4009

**Published:** 2014-01-17

**Authors:** Xin-Hong Xu, Wei-Min Qu, Min-Juan Bian, Fang Huang, Jian Fei, Yoshihiro Urade, Zhi-Li Huang

Figure 2 is incorrect. Please find a correct version of the figure here: http://plosone.org/corrections/pone.0047596.g002.cn.tif

